# Sleep duration, selected circulating biomarkers, and colorectal cancer risk

**DOI:** 10.1097/CEJ.0000000000000993

**Published:** 2026-03-25

**Authors:** Marta Rossi, Alessia Pauletto, Silvia Mignozzi, Paola Bertuccio, Konstantinos K Tsilidis, Massimiliano Mutignani, Marcello Cintolo, Irene Cottone, Roberto Penagini, Maurizio Vecchi, Michail Katsoulis, Simone Rampelli, Federica D’Amico, Giovanni Corso, Mirko Marino, Marco Rendine, Cristian Del Bo’, Patrizia Riso, Rossella Bonzi, Simone Guglielmetti, Carlo La Vecchia, Francesca Bravi

**Affiliations:** a Department of Clinical Sciences and Community Health, Dipartimento di Eccellenza 2023-2027, University of Milan, Milan; b Department of Public Health, Experimental and Forensic Medicine, University of Pavia, Pavia, Italy; c Department of Epidemiology and Biostatistics, School of Public Health, Imperial College London, London, UK; d Department of Hygiene and Epidemiology, University of Ioannina School of Medicine, Ioannina, Greece; e Digestive and Interventional Endoscopy Unit, Azienda Socio Sanitaria Territoriale (ASST) Grande Ospedale Metropolitano Niguarda; f Foundation IRCCS Ca’ Granda Ospedale Maggiore Policlinico; g Department of Pathophysiology and Transplantation, University of Milan, Milan, Italy; h Unit for Lifelong Health and Ageing, Institute of Cardiovascular Science, University College London, London, UK; i Unit of Microbiome Science and Biotechnology, Department of Pharmacy and Biotechnology, University of Bologna, Bologna, Italy; j Division of Breast Surgery, European Institute of Oncology IRCCS; k Department of Oncology and Hemato-Oncology, University of Milan, Milan; l Division of Human Nutrition, Department of Food, Environmental and Nutritional Sciences, University of Milan; m μbEat Lab, Department of Biotechnology and Biosciences (BtBs), University of Milano-Bicocca, Milan, Italy

**Keywords:** bacterial translocation, colorectal cancer, inflammation, leaky gut, sleep, zonulin

## Abstract

Sleep duration has been proposed to influence the risk of colorectal cancer (CRC). An involvement of inflammation, metabolic disorders, and gut permeability has been suggested. We investigated the relationship between sleep duration and CRC risk and examined whether sleep duration was associated with selected inflammatory and metabolic markers, and markers of gut permeability and bacterial translocation from the intestine to bloodstream. We used data from an Italian case-control study including 212 subjects (71 CRC cases and 141 tumor-free subjects). Sleep habits were collected through a questionnaire, including information on the average hours of sleep per night. We measured serum C-reactive protein (CRP) and glycemia by the ILab System, lipopolysaccharide-binding protein and zonulin by ELISA kit, and blood bacterial 16S rRNA gene copies by quantitative PCR and sequencing. We derived the odds ratios (OR) and corresponding 95% confidence intervals (CI) of CRC according to sleep duration from multiple logistic regression models. There was a positive association between long sleep duration and CRC risk, OR, 3.36 (95% CI, 1.08–10.53) for ≥ 9 compared to 7–8 h. For ≤ 6 h, the OR was 1.62 (95% CI, 0.84–3.29). BMI, circulating levels of CRP and glycemia, and a species of *Streptococcus* appeared to be higher in subjects reporting ≥9 vs. 7–8 h of sleep. Our data show a positive relationship between long sleep on CRC risk and suggest possible insights on inflammation, metabolic disorders, and possibly gut barrier dysfunction explaining this association.

## Introduction

Colorectal cancer (CRC) is one of the most common cancers worldwide, ranking third in terms of incidence and second in mortality (Bray *et al*., 2024). In 2022, there were about 1.9 million CRC cases and over 900 000 deaths globally (Bray *et al*., 2024). In Europe, predictions for 2025 estimate a mortality rate of about 14.5 deaths per 100 000 men and 7.9 per 100 000 women (Santucci *et al*., 2025).

Modifiable risk factors for CRC include tobacco smoking (Gram *et al*., 2020), alcohol consumption (Bagnardi *et al*., 2015), physical activity (Levi *et al*., 1999), selected aspects of diet (Schwingshackl *et al*., 2018), and overweight and obesity (Russo *et al*., 1998). In addition to these lifestyle behaviors, an increasing number of studies identified an association between sleep pattern, including sleep duration, quality and disorders, and CRC risk (Chen *et al*., 2018; Lin *et al*., 2019; Yoon *et al*., 2023). In particular, long (≥9 h), but not short (≤6 h), compared to normal (7–8 h) sleep duration was associated with 21% increase of CRC risk in a meta-analysis of six US cohort studies including about 8000 CRC cases (Hirshkowitz *et al*., 2015; Chen *et al*., 2018). Furthermore, long sleep duration was associated with 10% CRC risk increase in the Multiethnic Cohort (MEC), including about 6000 CRC cases (Tembo *et al*., 2025) and 59% CRC risk increase in a case–control study from Spain, including about 2800 CRC cases (Papantoniou *et al*., 2017). However, the evidence on the association between sleep duration and CRC remains inconclusive (McNeil *et al*., 2020), and a possible effect of reverse causation should be considered (Papantoniou *et al*., 2021; Liu *et al*., 2025).

There are possible biological mechanisms supporting a role of sleep duration on CRC risk (Liu *et al*., 2025). Long sleep duration has been linked to pro-inflammatory processes, as marked by C-reactive protein (CRP) levels (Grandner *et al*., 2013; He *et al*., 2020), impaired glucose metabolism (Jang *et al*., 2023), and obesity (Xu *et al*., 2025), which play a role in CRC development (Russo *et al*., 1998; Mutignani *et al*., 2021; Pearson-Stuttard *et al*., 2021; Liu *et al*., 2025). Sleep disorders can alter gut microbiota composition and promote dysbiosis, leading to impaired intestinal barrier function (Marino *et al*., 2024). Dysbiosis has been associated with an increased intestinal permeability or ‘leaky gut’, which reflects the intestine’s ability to regulate what enters the bloodstream (Camilleri, 2019). An increased permeability can promote bacterial translocation from the gut into the circulation (Mutignani *et al*., 2021), potentially contributing to both the development and progression of CRC (Liu *et al*., 2025).

This study aims to investigate the relationship between sleep duration and CRC risk. We also explore whether sleep duration can be linked to inflammatory and metabolic markers such as CRP, glycemia, body mass index (BMI), as well as markers of gut permeability and bacterial translocation, such as serum zonulin, lipopolysaccharide-binding protein (LBP), blood bacterial 16S rRNA gene copies, and selected bacterial taxa.

## Methods

### Study design, setting, and sample

We analyzed data from a case–control study conducted in two university hospitals of Milan, Italy, between 2017 and 2019 (Mutignani *et al*., 2021). Subjects were recruited among patients aged 20–85 years, scheduled for a colonoscopy in one of the two hospitals. We applied the following exclusion criteria: immunodeficiency, selected inflammatory diseases, liver/kidney/heart failure, reported previous cancer, and recent hospitalization or colonoscopy. Two pathologists reviewed colonoscopies and histological examinations to identify cases of incident, histologically confirmed CRC. Controls include subjects with intestinal adenoma (IA) or subjects free from IA/CRC. Each CRC case was matched to one IA and one subject free from IA/CRC by study center, sex, and age (± 5 years).

The study was conducted according to the guidelines of the Declaration of Helsinki, and the protocol was approved by the ethical committees of Fondazione IRCCS Ca’ Granda Ospedale Maggiore Policlinico (No. 742–2017; 14 December 2017) and Azienda Socio Sanitaria Territoriale Grande Ospedale Metropolitano Niguarda (No. 477–112016; November 25, 2016). Less than 2% of contacted subjects declined participation. All the enrolled subjects signed a written informed consent, and a total of 347 subjects were recruited.

This analysis includes subjects with available serum samples: 71 CRC cases and 141 controls.

### Data collection

All subjects were interviewed by trained interviewers, blinded to the CRC status, using a questionnaire including sociodemographic, education, lifestyle habits (e.g. occupational physical activity), and anthropometric (e.g. weight and height) data. We collected information on the use of drugs at least once a week for more than 6 months, including aspirin use. The questionnaire comprised a section on sleep habits before the diagnosis, including information on the usual time at which the subject falls asleep, the average hours of sleep per night, and sleep disorders. Responses related to sleep duration were provided in half-hour increments, ranging from 3 to 12 h per night.

Blood samples were collected in 7 mL EDTA tube and in 3 mL blank tube before colonoscopy. Part of the EDTA sample was stored at −80 °C for bacterial DNA analyses. The sample without anticoagulant was processed and centrifuged (1400 g × 15 min, 4 °C) to obtain serum samples, and then stored at −80 °C.

### Computation of serum C-reactive protein, glycemia, lipopolysaccharide-binding protein, zonulin, and blood bacterial 16S rRNA gene copies

CRP and glycemia were measured in serum using the ILab System by Werfen Company (Barcelona, Spain). The method for CRP used polystyrene latex particles of uniform size coated with IgG antihuman CRP. Glucose was determined using an endpoint based on the Trinder reaction, employing glucose oxidase and peroxidase.

LBP concentrations in serum were quantified using a commercial ELISA kit (Cat. #EH1560, Wuhan Fine Biotech Co., Ltd., Wuhan, China). Serum aliquots were thawed once at room temperature before analysis. The assay is based on a sandwich enzyme-linked immunosorbent technique, employing a microplate precoated with anti-LBP antibodies. Samples and standards were incubated at 37 °C, followed by the addition of a biotin-labelled detection antibody and an HRP-conjugated streptavidin solution. After the enzymatic reaction with Tetramethylbenzidine (TMB) substrate, absorbance was recorded at 450 nm using a microplate reader (mod. Infinite F200, Tecan, Milan, Italy). LBP concentrations were calculated from a standard curve fitted using a four-parameter logistic model.

Serum zonulin levels were quantified using the ELISA kit (cat. # K5601) from Immunodiagnostik (Bensheim, Germany) (Marino *et al*., 2024). The 96-well plate was precoated with a polyclonal antizonulin antibody. A biotinylated zonulin tracer was added to each sample, and the peroxidase-labelled streptavidin was added to bind the biotinylated zonulin tracer. The reading of the fluorescence at 450 nm was performed using a TECAN Infinite F200 plate reader. Zonulin levels were quantified using a standard curve calculated by a 4-parameter algorithm.

For 16S rRNA gene copies analyses, DNA extraction, quantitative PCR (qPCR) experiments, and sequencing of 16S rRNA gene amplicons were performed by Vaiomer SAS (Labège, France) through an optimized blood-specific technique (Mutignani *et al*., 2021). In particular, we obtained data on the presence and the absolute abundances of family *Enterobacteriaceae*, genus *Streptococcus*, and an undefined species of the *Streptococcus* genus.

For the computation of all biomarkers, operators were blinded to the CRC status.

### Statistical analysis

We assessed the relationship between sleep duration and CRC risk using logistic regression models, including terms for study center, sex, age, education, occupational physical activity, BMI, and aspirin use. We computed the odds ratios (OR) of CRC and the corresponding 95% confidence intervals (CI) for a sleep duration of ≥9 and for ≤6 vs. 7–8 h. We further adjusted for alcohol consumption, smoking habits, red meat consumption, and diabetes.

A restricted cubic spline logistic model with four knots was used to show the relationship between sleep duration in hours per night and CRC risk, using 8 h as the reference group (Desquilbet and Mariotti, 2010; Harrell, 2017). The four knots were at the 5th, 35th, 65th, and 95th percentiles, corresponding to 5, 7, 8, and 9 h of sleep. The model was consistent with the core model. Nonlinearity was assessed through a likelihood-ratio test comparing models with and without the restricted cubic spline term.

We investigated inflammatory and metabolic markers such as CRP, glycemia and BMI, as well as markers of gut permeability and bacterial translocation such as zonulin, LBP, bacterial 16S rRNA gene copies, and selected taxa by sleep duration (≤ 6, 7–8, ≥ 9 h) overall and among controls, through two-tailed Kruskal–Wallis test and Wilcoxon rank sum test. The heterogeneity of the presence of selected taxa according to sleep duration was estimated through a $\chi_{2}^{2}$ test. Violin plots display the distributions of CRP, glycemia, BMI, zonulin, LBP, and 16S rRNA gene copies by sleep duration.

We evaluated whether the frequency of subjects reporting ≤ 6, 7–8, and ≥ 9 h of sleep was different across strata of CRP (<3 and ≥ 3 mg/L), glycemia (<100 and ≥ 100 mg/dL) and BMI (<25 and ≥ 25 kg/m²) through a $\chi_{1}^{2}$ test.

## Results

CRC cases were 71 (45 males, 63.4%), and controls were 141 (92 males, 65.3%; *P* for $\chi_{1}^{2}$: 0.65). The two groups had a similar distribution in terms of study center, age, education, occupational physical activity and BMI.

**Fig. 1:**
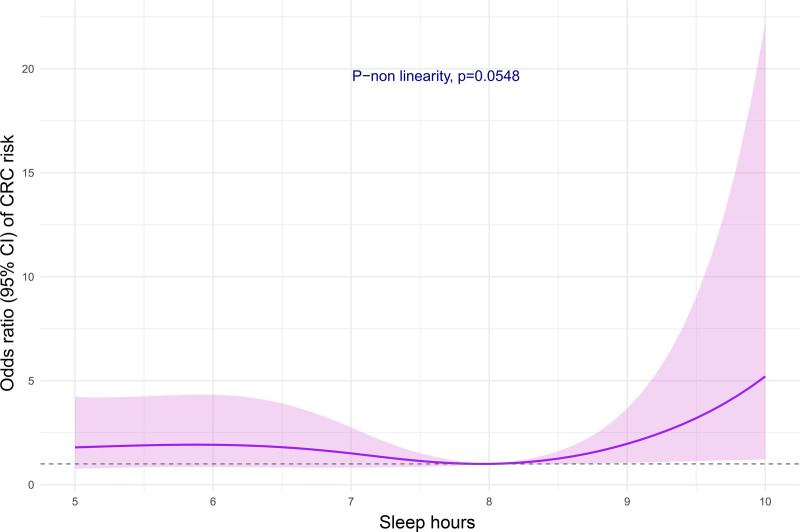
Odds ratios from logistic regression models with restricted cubic splines curves describing the association between sleep duration and colorectal cancer (CRC) risk. The horizontal line represents an odds ratio of 1. Milan, Italy, 2017–2019.

Table 1 gives the distribution of CRC cases and controls according to sleep duration (≤ 6, 7–8, and ≥ 9 h) and the corresponding OR and 95% CI. Compared to 7–8 h of sleep, the OR of CRC was 3.36 (95% CI, 1.08–10.53) for subjects sleeping ≥9 h and 1.62 (95% CI, 0.84–3.29) for those sleeping ≤6 h. The ORs did not change after further adjusting for alcohol consumption, smoking habits, red meat consumption, and diabetes. Figure 1 suggests a nonlinear relationship between hours of sleep and CRC risk (*P* for nonlinearity test: 0.055) using 8 h as the reference group. The curve was steeper above 8 h of sleep than below, with significant association above and – not below – 8 h. Specifically, the ORs of CRC were 1.97 (95% CI, 1.06–3.66) for 9 h and 5.21 (95% CI, 1.22–22.22) for 10 h as compared with 8 h.

**Table 1:** Distribution, odds ratios and corresponding 95% confidence intervals of colorectal cancer risk for sleep duration among 71 colorectal cancer cases and 141 controls. Milan, Italy, 2017–2019

	n (%)		OR^a^
Sleep duration (h)	CRC cases	Controls	(95% CI)
≤6	22 (31.0%)	34 (24.1%)	1.62
			(0.84–3.29)
7–8	41 (57.7%)	100 (70.9%)	1 (ref)
≥9	8 (11.3%)	7 (5.0%)	3.36
			(1.08–10.53)

CRC, colorectal cancer; CI, confidence interval; OR, odds ratio.

a Estimated from a logistic regression model including study center, age, sex, education, occupational physical activity, body mass index and aspirin use.

Table 2 presents the median and quartiles I and III of inflammatory and metabolic markers, as well as markers of gut permeability and bacterial translocation, by subjects reporting ≤ 6, 7–8, and ≥9 h of sleep and the corresponding test for heterogeneity. Individuals with long sleep duration had higher levels of inflammatory and metabolic markers (CRP, glycemia, and BMI) and of LBP and a species of genus *Streptococcus*, with significant difference only for the last one (*P*: 0.044). Moreover, the prevalence of this species was significantly higher in subjects with a sleep duration of ≥9 (46.7%) and ≤6 (39.3%) than 7–8 h (24.1%) (*P* for heterogeneity: 0.036) (data not shown). No differences in terms of other biomarkers were found between ≤ 6 and 7–8 h of sleep. Among the control group, glycemia levels were significantly higher in individuals sleeping ≥9 (median: 109.0 mg/dL) than in those sleeping 7–8 h (median: 88.0 mg/dL) (*P*: 0.02) (Supplementary Table 1, Supplemental digital content 1, https://links.lww.com/EJCP/A574). Supplementary Figure 1 A and B (Supplemental digital content 1, https://links.lww.com/EJCP/A574) show violin plots of these markers suggesting a different shape of distributions according to ≥9 vs. 7–8 h of sleep.

**Table 2:** Median, I – III quartile, and p-value comparison of selected biomarkers by sleep duration. Milan, Italy, 2017–2019

	Median (I–III Q)					
Sleep duration (h)	≤ 6	7 - 8	≥ 9	P* 7–8 vs. ≤6	P* 7–8 vs. ≥9	P* for Kruskal–Wallis
Inflammatory and metabolic markers						
CRP	3.0 (2.0–4.9)	3.3 (2.4–6.1)	4.7 (3.2–7.8)	0.15	0.19	0.11
Glycemia	85.0 (76.0–102.0)	88.0 (75.0–99.0)	94.0 (83.0–112.0)	0.92	0.14	0.32
BMI	25.1 (22.8–27.0)	24.8 (23.4–27.0)	26.4 (23.6–29.7)	0.90	0.14	0.31
Markers of gut permeability and bacterial translocation						
Zonulin	30.0 (27.0–33.3)	29.3 (26.3–32.4)	29.7 (27.8–32.6)	0.27	0.23	0.31
LBP	59.8 (24.4–157.9)	69.2 (32.2–182.4)	155.8 (44.5–175.6)	0.22	0.26	0.19
16S rRNA gene copies	6888.9 (5504.2–9072.6)	7342.0 (5655.0–9416.3)	6959.0 (6047.3–9851.6)	0.30	0.76	0.57
Family *Enterobacteriaceae* (abundances)	38.2 (0.3–210.3)	27.3 (0.3–284.7)	0.7 (0.3–53.4)	0.98	0.28	0.53
Genus *Streptococcus* (abundances)	0.12 (0.0–107.51)	0.00 (0.00–0.31)	0.00 (0.00–111.70)	0.04	0.38	0.11
Species of *Streptococcus* (undefined) (abundances)	0.00 (0.00–0.29)	0.00 (0.00–0.00)	0.00 (0.00–69.92)	0.02	0.04	0.02

CRP, C-reactive protein; BMI, body mass index; LBP, lipopolysaccharide-binding protein.

* *P* for heterogeneity.

Table 3 shows that the proportion of subjects reporting ≥9 comparing to 7–8 h of sleep was higher in CRP ≥ 3 (13.5%) than <3 mg/L (3.3%) (*P*: 0.035), in glycemia ≥100 (17.9%) than <100 mg/dL (6.8%) (*P*: 0.042), and in BMI ≥ 25 (13.3%) than <25 kg/m² (6.2%) (*P*: 0.127). Similar distributions were found for sleep duration of ≤6 and 7–8 h.

**Table 3:** Distribution of C-reactive protein, glycemia, and BMI according to sleep duration. Milan, Italy, 2017–2019

	*n* (%)									
	CRP (mg/L)		*P* for $\chi_{1}^{2}$		Glycemia (mg/dL)		*P* for $\chi_{1}^{2}$	BMI (kg/m²)		*P* for $\chi_{1}^{2}$
	<3	≥3			<100	≥100		<25	≥25	
Sleep duration (hours)										
7–8	58 (96.7%)	83 (86.5%)	0.035	109 (93.2%)		32 (82.0%)	0.042	76 (93.8%)	65 (86.7%)	0.127
≥9	2 (3.3%)	13 (13.5%)		8 (6.8%)		7 (18.0%)		5 (6.2%)	10 (13.3%)	
Total	64	92		117		39		81	39	

CRP, C-reactive protein; BMI, body mass index.

## Discussion

This study found an elevated risk of CRC for subjects reporting 9 or more than 7–8 h of sleep, suggesting that long sleep might play a role in CRC development. Subjects reporting less than 7 h of sleep also had an increased, though not significant, CRC risk. Long sleep appeared to be unfavorable in terms of inflammatory, metabolic, and possibly gut permeability markers.

Our findings are in line with data from the literature showing a direct association between long sleep duration and CRC risk (Papantoniou *et al*., 2017; Chen *et al*., 2018; Tembo *et al*., 2025). A J-shaped association (with a significant nonlinear trend) between sleep duration and CRC risk was reported in a meta-analysis, with the lowest CRC risk at a sleep duration of 7 h per night (Chen *et al*., 2018). We found a similar shape of association with the lowest CRC risk at a sleep duration of 8 h per night, and with a significant risk increase above 8 h. A morning chronotype, which may be inversely linked to long sleep, was associated with a reduced risk of CRC in a Mendelian randomization analysis on the UK Biobank and FinnGen data (Yuan *et al*., 2023), and on data from three large CRC consortia among men (Dimopoulou *et al*., 2025), revealing a possible influence of sleep duration on CRC. Long sleep was also associated with increased CRC mortality in the Cancer Prevention Study-II in men (Donzella *et al*., 2024). However, no association between sleep duration and CRC mortality was found in a previous systematic review, either in men or in women (Stone *et al*., 2019).

The mechanisms by which long sleep influences CRC risk can be related to systemic inflammation, metabolic conditions, and gut microbiota dysfunction. Long sleep duration has been associated with elevated levels of CRP (Patel *et al*., 2009) – in line with our data – and of pro-inflammatory cytokines (IL-6), which can promote CRC (Li *et al*., 2020). Abnormal sleep patterns and durations may affect the composition and functionality of gut microbiota via the brain-gut-microbiota axis through neuroendocrine, hypothalamic-pituitary-adrenal axis, immune, and neural pathways (Han *et al*., 2022). The vagus nerve connects gut and brain function through signals from intestinal immune cells, bacterial metabolites such as short-chain fatty acids (SCFAs) and neurotransmitters like γ-aminobutyric acid. These exhibit intestinal epithelial preservation properties and antiinflammatory activities against CRC (Liu *et al*., 2021). Lower fecal SCFA levels and circulating biomarkers of gut impairment have been associated with an increased risk of intestinal adenoma and CRC (Alvandi *et al*., 2022; Seethaler *et al*., 2022; Shi *et al*., 2023; Marino *et al*., 2024). In line with a possible involvement of Gram-negative bacteria in fatigue and increased sleep duration related to intestinal permeability and inflammation (Maes *et al*., 2007; Szentirmai *et al*., 2024), we found higher – although not significant – levels of LBP in subjects with long compared to normal sleep duration. We found no differences in terms of sleep duration for Gram-negative bacteria such as Enterobacteriaceae, which are abundant in gut dysbiosis (Moreira de Gouveia *et al*., 2024). Instead, we found significantly higher presence and abundance of a species belonging to *Streptococcus* in subjects with long as compared with normal sleep duration (*P* for heterogeneity: 0.044 and 0.036, respectively). This may reflect a distinct mechanism of bacterial translocation compared with LBP, as *Streptococcus* is a Gram-positive bacterium, and its translocation involves different immune pathways (toll-like receptor, TLR2 vs. TLR4). An *in vivo* study found a link between immune activation mediated by peptidoglycan components of Gram-positive bacteria and increased fatigue and sleepiness (Szentirmai *et al*., 2021).

Although there is evidence of a complex interplay between sleep duration, gut barrier dysfunction, and systemic immune activation, it remains unclear which one is responsible for initiating the process, since gut dysbiosis in turn can contribute to increase inflammation and sleep disorders. Subjects reporting long sleep duration appeared to have higher levels of CRP, glycemia, and higher BMI in our data. Moreover, they had more frequently medium/high than normal CRP levels, prediabetic/diabetic than nondiabetic conditions, and overweight/obesity than normal/underweight, as compared with those reporting normal sleep duration. This is in line with evidence linking long sleep duration to increased BMI and obesity, possibly through reduced energy expenditure, hormonal dysregulation, and low-grade inflammation (Liu *et al*., 2019). Prolonged sleep could lead to metabolic dysregulation, such as insulin resistance and alterations in appetite-regulating hormones, leading to increased appetite, hyperglycemia, and subsequent obesity (Liu *et al*., 2025). Sleep habits, eating behavior, including overconsumption and quality of food (especially before sleeping) and prolonged periods of inactivity are all relevant factors and may contribute to inflammatory and metabolic events through a synergic effect (Zhao *et al*., 2020; Hepsomali and Groeger, 2022). Metabolic disturbances can affect levels of tryptophan, serotonin, and, in turn, melatonin, which is involved in immune regulation, apoptosis, and proliferation of CRC cells (Liu *et al*., 2025).

Although the evidence from epidemiological studies is less clear for short than long sleep (Chen *et al*., 2018; Tembo *et al*., 2025), a J-shaped relationship between sleep duration and CRC risk highlights potential detrimental effects of short sleep on CRC risk, as well. Some mechanisms in supporting the positive association with CRC risk are the same as long sleep, including those related to alterations of appetite-regulating hormones (van Egmond *et al*., 2023; Tembo *et al*., 2025). Whereas an increased risk of CRC for long sleep appears to be accompanied by an increase in metabolic markers, short sleep was not associated with these markers in our data. The MEC study found a 35% increased CRC risk for short sleep combined with obesity (Tembo *et al*., 2025), suggesting that obesity may act as a modifier amplifying the adverse effect of insufficient sleep duration on CRC risk. Sleep deprivation has also been suggested as a key factor in explaining the link between nightshift work and cancer risk (Esposito *et al*., 2025).

### Study limitations

With reference to possible selection bias, the data were derived from an ad-hoc data collection in which cases and controls were recruited from the same catchment areas. Exclusion criteria considered chronic conditions related to the study hypothesis for both cases and controls. Cases were detected at the first CRC-diagnosing colonoscopy, minimizing the time between recruitment and diagnosis, as well as the possibility of lifestyle changes occurring in the recent past. Controls include a portion of subjects with IA. However, when excluding IA from controls, results were virtually identical. Information bias was reduced since the questionnaire was administered by trained and blinded interviewers before colonoscopy; cases were unaware of the diagnosis at the time of the interview, virtually eliminating the misreporting by cases. The questionnaire included a section on sleep habits, including different aspects (e.g. sleep disorders and bedtime), and sleep information other than duration did not show any significant results in our data. We were not able to provide standardized measures of sleep quality (e.g. Pittsburgh Sleep Quality Index). However, we collected information on hours of sleep from all subjects and categorized sleep exposure in three duration categories according to the recommendations of the American Sleep Foundation, which defined optimal sleep as 7–8 h per night (Watson *et al*., 2015). Other sleep traits may influence our results, although most subjects with long sleep duration did not report any sleep disorders. No information on daytime napping and obstructive sleep apnea was available in our data. With reference to confounding, we were able to adjust for several factors, such as BMI, lifestyle habits, including dietary aspects, and medical conditions. However, other modifiable factors, including mental disorders, may influence sleep duration (also through gut microbiota changes) and CRC risk, directly or indirectly (Chen *et al*., 2018; Zhang *et al*., 2022). A possible reverse causation should also be considered since CRC may lead to the occurrence of common symptoms, including weakness and fatigue, which may affect sleep duration (Crowder *et al*., 2024). However, when cases with metastasis were excluded, as they may have had the tumor for longer than those in stages I–III, the results remained unchanged. Another limitation is the small sample size, which can limit multivariate analysis and causal inference. However, our results were in line with most literature (Keum and Giovannucci, 2019; Speciani *et al*., 2022, 2023; Mignozzi *et al*., 2025). Larger studies would allow more in-depth statistical analyses, such as mediation analyses, to investigate the causes underlying colorectal carcinogenesis.

### Conclusion

Our findings provide additional evidence supporting an association between long sleep and CRC risk and suggest that mechanisms related to inflammation and metabolic disorders – and possibly gut impairment – can explain this association. Further evidence from large-scale longitudinal studies is needed to quantify this association and to evaluate whether sleep patterns should be integrated into CRC prevention strategies alongside established lifestyle risk factors.

## Acknowledgements

The authors express their gratitude to all participants and collaborators to this study, without whose effort this work would not have been feasible. A special thanks to Margherita Cozzi for her valuable involvement in this study. We thank Clorinda Ciafardini, Elena Tansi, Cinzia Della Noce, Rosa Restieri, Nadia Zaretti, as well as all the nursing staff at the Digestive and Interventional Endoscopy Unit, ASST Grande Ospedale Metropolitano Niguarda, Milan, and at the Gastroenterology and Endoscopy Unit, Fondazione IRCCS Ca’ Granda Ospedale Maggiore Policlinico, Milan. A thankful mention to Luisa De Simone e Giuseppe Giovenzana for their constant and accurate help in the preparation of the laboratory material. Lastly, we would like to express our sincere thanks to Cinzia Delorenzi and Barbara De Pasquale for the insights and suggestions they contributed to this issue.

Data collection was supported by the Italian Foundation for Cancer Research (AIRC) (My First AIRC grant No. 17070) (MR). Data analysis was supported by the grant PRIN 2022 PNRR (no. P20229A9S5) from the Italian Ministry of University and Research (MUR). The work of GC was partially supported by the Italian Ministry of Health 5 × 1000 funds Ricerca Corrente.

### Conflicts of interest

There are no conflicts of interest.

## Supplementary Material
