# Supplementary material for: Sleep duration, selected circulating biomarkers, and colorectal cancer risk
Source: Eur J Cancer Prev. 2026 Mar 25;35(3):258–65. doi: 10.1097/CEJ.0000000000000993 (PMC13011957; doi:10.1097/CEJ.0000000000000993)

**Supplementary Table 1**. Median, I – III quartile, and p-value comparison of selected biomarkers by sleep duration in the control group. Milan, Italy, 2017-2019.

|  |  | Median  (I – III Q) | | | p*  7 – 8 vs. ≤ 6 | p*  7 – 8 vs. ≥ 9 | p*  for Kruskal-Wallis |
| --- | --- | --- | --- | --- | --- | --- | --- |
| Sleep duration (hours) | | ≤ 6 | 7 - 8 | ≥ 9 |  |  |  |
| Inflammatory and metabolic markers | | | |  |  |  |  |
|  | CRP | 2.7  (2.0 – 4.3) | 3.1  (2.4 - 5.5) | 4.7  (3.1 - 7.8) | 0.16 | 0.29 | 0.17 |
|  | Glycemia | 84.5  (73.0 – 103.0) | 88.0  (75.5 - 99.5) | 109.0  (90.0 - 138.0) | 0.63 | 0.02 | 0.06 |
|  | BMI | 24.7  (22-6 – 26.5) | 24.9  (23.4 - 27.4) | 26.4  (25.4 - 29.3) | 0.53 | 0.13 | 0.24 |
| Markers of gut permeability and bacterial translocation | | | | |  |  |  |
|  | Zonulin | 29.4  (26.9 – 32.3) | 28.6  (26.0 - 31.6) | 29.7  (27.4 - 31.4) | 0.40 | 0.38 | 0.51 |
|  | LBP | 38.6  (23.2 – 114.3) | 60.0  (27.6 - 164.6) | 125.5  (37.4 - 163.7) | 0.22 | 0.64 | 0.36 |
|  | 16S rRNA gene copies | 7286.8  (5638.8 - 9041.0) | 7205.7  (5630.4 - 9124.2) | 7104.1  (6047.3 - 10576.3) | 0.97 | 0.64 | 0.88 |
|  | Family *Enterobacteriaceae* | 41.8  (0.2 – 182.0) | 24.8  (0.3 – 269.4) | 1.3  (0.5 – 96.2) | 0.61 | 0.65 | 0.81 |
|  | Genus *Streptococcus* | 0.11  (0.0 – 170.04) | 0.00  (0.00 – 0.45) | 0.00  (0.00 – 313.24) | 0.32 | 0.73 | 0.60 |
|  | Species of *Streptococcus* (undefined) | 0.00  (0.00 – 0.21) | 0.00  (0.00 – 0.04) | 0.00  (0.00 – 0.32) | 0.20 | 0.33 | 0.32 |

* p for heterogeneity

**Supplementary Figure 1 (A)**. Violin Plot of C-reactive protein (CRP), glycemia, and body mass index (BMI) by sleep duration. Milan, Italy, 2017-2019.

**
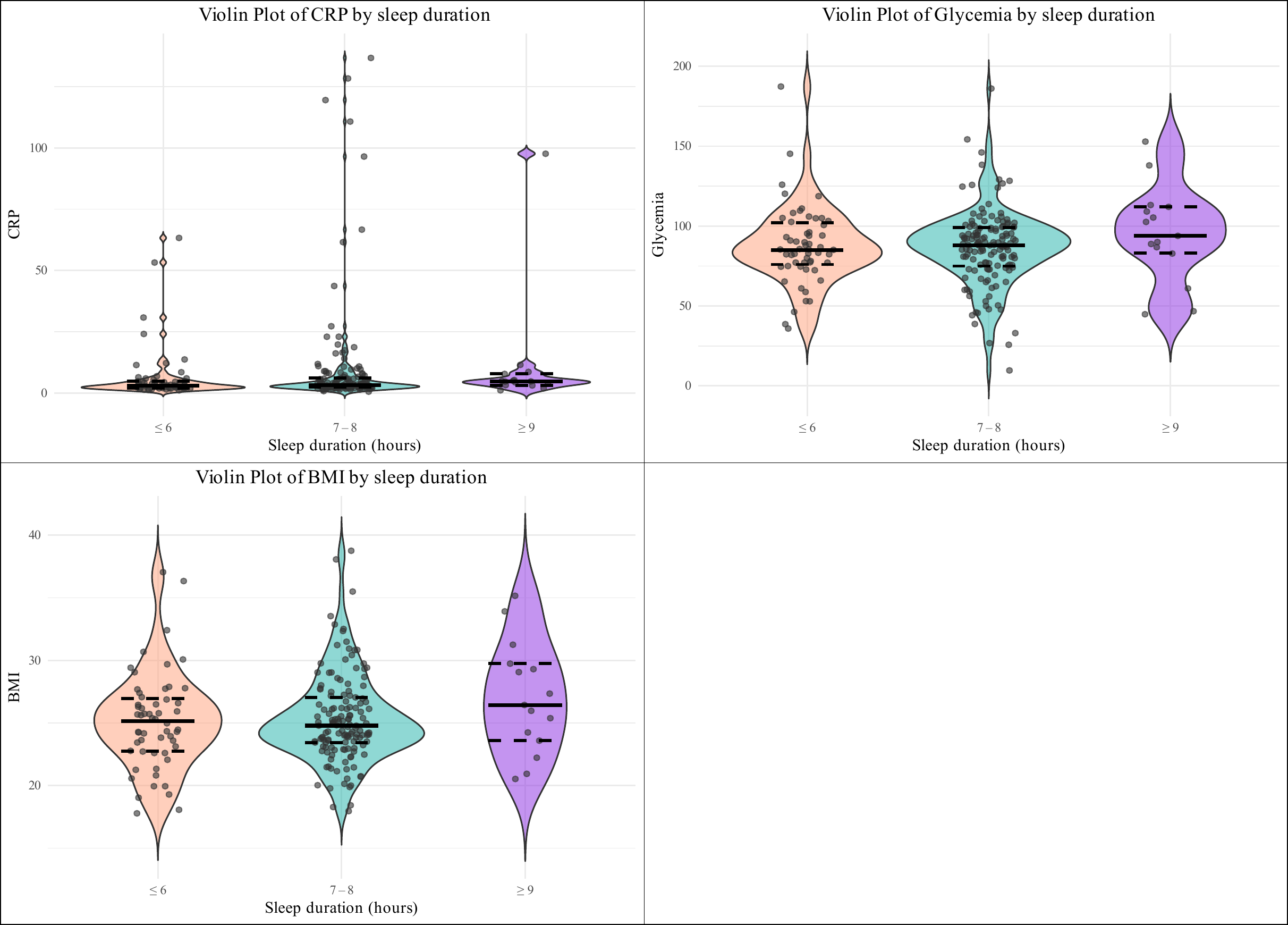
**

**Supplementary Figure 1 (B)**. Violin Plot of serum zonulin, lipopolysaccharide-binding protein (LBP), blood bacterial 16s rRNA gene copies and a species of genus streptococcus by sleep duration. Milan, Italy, 2017-2019.


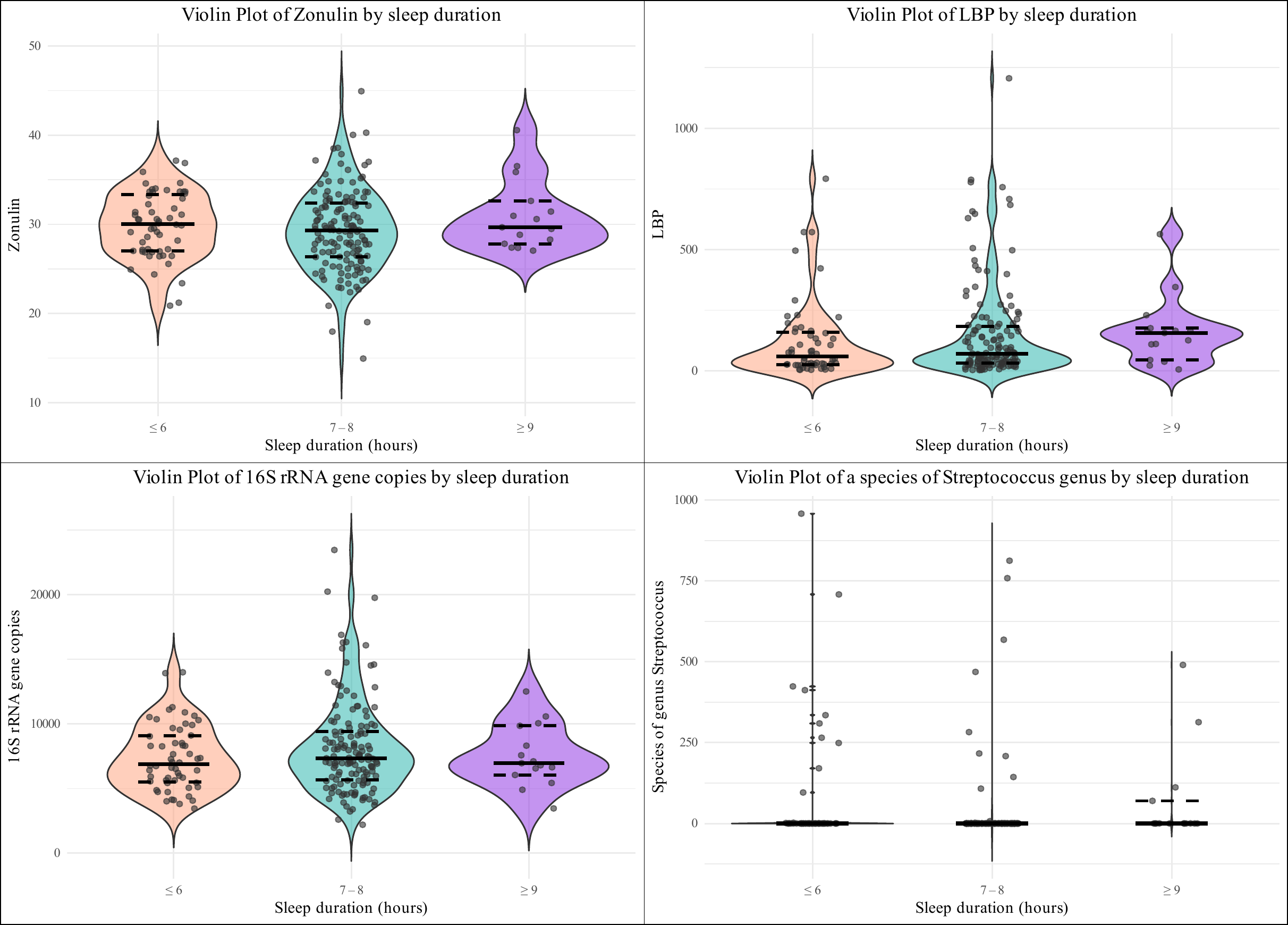

Supplement: Supplementary file 1 [file ejcp-35-258-s001.docx]
